# Memory or acclimation of water stress in pea rely on root system's plasticity and plant's ionome modulation

**DOI:** 10.3389/fpls.2022.1089720

**Published:** 2023-01-25

**Authors:** Cécile Jacques, Sylvie Girodet, Fanny Leroy, Sylvain Pluchon, Christophe Salon, Marion Prudent

**Affiliations:** ^1^ Agroécologie, INRAE, Institut Agro, Univ. Bourgogne, Univ. Bourgogne Franche-Comté, Dijon, France; ^2^ Plateforme PLATIN’, US EMerode, Normandie Université, Unicaen, Caen, France; ^3^ Laboratoire de Nutrition Végétale, Centre Mondial de l’Innovation Roullier, TIMAC AGRO, Saint Malo, France

**Keywords:** legume, water deficit, recurrent stress, nutrient uptake, agroecology

## Abstract

**Introduction:**

Peas, as legume crops, could play a major role in the future of food security in the context of worldwide human nutrient deficiencies coupled with the growing need to reduce consumption of animal products. However, pea yields, in terms of quantity and quality (i.e. grain content), are both susceptible to climate change, and more specifically to water deficits, which nowadays occur more frequently during crop growth cycles and tend to last longer. The impact of soil water stress on plant development and plant growth is complex, as its impact varies depending on soil water availability (through the modulation of elements available in the soil), and by the plant’s ability to acclimate to continuous stress or to memorize previous stress events.

**Method:**

To identify the strategies underlying these plant responses to water stress events, pea plants were grown in controlled conditions under optimal water treatment and different types of water stress; transient (during vegetative or reproductive periods), recurrent, and continuous (throughout the plant growth cycle). Traits related to water, carbon, and ionome uptake and uses were measured and allowed the identification typical plant strategies to cope with water stress.

**Conclusion:**

Our results highlighted (i) the common responses to the three types of water stress in shoots, involving manganese (Mn) in particular, (ii) the potential implications of boron (B) for root architecture modification under continuous stress, and (iii) the establishment of an “ecophysiological imprint” in the root system *via* an increase in nodule numbers during the recovery period.

## 1 Introduction

Drought is one of the most impacful environmental factors that impairs plant growth, development, and, finally, plant yields (Prasad et al., 2008; Lipiec et al., 2013; Conforti et al., 2018). In the context of climate change, crops face frequent and severe stressful conditions during their growth cycle; among these is the higher frequency of drought events. Nowadays, drought events tend to occur earlier in the crop growth cycle, in the spring period, whereas in previous years they tended to occur at later stages of the growth cycle (Hänsel et al., 2019).

Drought decreases photosynthetic activity (Zhou et al., 2007) either by way of stomatal closure (Chaves, 1991; Flexas et al., 2004) or directly, negatively impacting metabolic activities (Farquhar et al., 1989; Parry, 2002; Bota et al., 2004). In parallel, a decrease of carbon dioxide (CO_2_) uptake or internal resistance to CO_2_ diffusion can be responsible for an increase in photorespiration (Wingler et al., 2000; Wingler et al., 2002; Prasad et al., 2008). Drought also induces morphological changes; these include a reduction in leaf expansion and size (Alves and Setter, 2004), which result from both a lower number of cells and smaller cells, which generates in turn a lower transpiration area (Randall and Sinclair, 1988; Tardieu et al., 2000; Basu et al., 2016). Drought-induced stomatal closure also lowers plant transpiration and water loss through leaves (Sobeih et al., 2004; Liu et al., 2006; Chai et al., 2016). These responses depend upon the extent of drought. In mild drought, both the number of leaves and the rate of leaf expansion are reduced; in severe drought, leaf growth cessation may occur (Prasad et al., 2008), and a prolongated period of drought may even accelerate leaf senescence (De Souza et al., 1997; Jagadish et al., 2015). Furthermore, roots, being the first organs perceiving soil water stress (Brunner et al., 2015; Weemstra et al., 2016), are also greatly impacted by water availability (Chaves, 1991; Zhou et al., 2007; Prasad et al., 2008; Chapman et al., 2012); under moderate water stress, carbohydrates partitioning to roots is maintained or increased, promoting their growth, but this is, by contrast, reduced by severe drought (Sharp and Davies, 1989; Wu and Cosgrove, 2000; Prasad et al., 2008; Prudent et al., 2016).

Lower soil water availability also reduces the nutrient availability for plants, which in turn limits their nutrient uptake and assimilation (Prasad et al., 2008; Gregory et al., 2012; Barzana et al., 2021). Moreover, water stress negatively impacts the transport of elements within plants owing to the decrease of transpiration, transporter activity, and cell membrane permeability (Viets, 1972; Hsiao, 1973; Kramer and Boyer, 1995; Sánchez-Rodríguez et al., 2010; Ahanger et al., 2016). Thus, under water stress, a nutritional imbalance of the plant ionome can be observed, which results from lower concentrations of essential elements for plant development and growth: macronutrients [nitrogen (N), sulfur (S), phosphorous (P), magnesium (Mg), calcium (Ca), and potassium (K)] or micronutrients [iron (Fe), manganese (Mn), copper (Cu), zinc (Zn), nickel (Ni), molybdenum (Mo), boron (B), and chlorine (Cl)] (Kirkby, 2012). Moreover, the availability of elements considered to be “beneficial” [(cobalt (Co), sodium (Na), vanadium (V), aluminium (Al), selenium (Se), and silicon (Si)] because they can improve plant development and growth and plant response to abiotic stresses may be reduced within water-stressed soils. However, interactions among ionome mineral elements complexifies the assessment of which ones are first impacted by water stress (Baxter et al., 2008; Salt et al., 2008). For example, the reduced availability of Fe can increase the uptake of other metal cations (Ni, Cu, Mn, and Zn), as these elements share the common transporter Iron-Regulated Transporter 1 (IRT1) (Pilon et al., 2009). If rewetting occurs after water stress, such a “recovery period” could trigger a higher level of soil mineralization that enhances nutrient availability for plants (Austin et al., 2004; He and Dijkstra, 2014; Wang et al., 2017).

Under low soil mineral nitrogen availability, legumes such as peas (*Pisum sativum* L.) mainly rely for their nitrogen nutrition on the symbiotic atmospheric N_2_ fixation, which is decreased by water stress *via* its negative impact on both structural and functional components (Zahran, 1999; Salon et al., 2001; Prudent et al., 2016). Indeed, drought decreases nodulation initiation, nodule growth, and nodule specific activity (King and Purcell, 2001; Serraj et al., 2001; Streeter, 2003; Larrainzar et al., 2009; Mahieu et al., 2009; Prudent et al., 2016). Altogether, this leads to a decrease in plant nitrogen levels and overall plant growth.

During long periods of continuous stress, plant acclimation includes reprogramming of development, physiology, and metabolism to improve plant functioning and promote better plant health (Lichtenthaler, 1996; Bohnert and Sheveleva, 1998; Orcutt et al., 2000; Wilson and Franklin, 2002; Sanchez et al., 2011). During their acclimation to periods of continuous stress, plants decrease their organ growth. Although early responses to water stress inlcude an induction of ABA signaling leading to stomatal closure (Harb et al., 2010; Pirasteh-Anosheh et al., 2016), the plant response evolves after some days, entering an intermediate stage that includes the regulation of cell wall properties (Neumann, 1995; Moore et al., 2008; Harb et al., 2010). Changes in cell walls, which are essential for plant adaptation (Bacon, 1999), seem to be linked to an early induction of expansin genes that are repressed during continuous stress (Jones and McQueen-Mason, 2004; Harb et al., 2010). Finally, during the later stages of periods of continuous moderate water stress, plant growth is so reduced that it enables the maintenance of plant metabolism and physiology at the same level as control plants as a result of a reduced energy demand (Harb et al., 2010). Indeed, acclimation was shown to maintain stomatal conductance and photosynthesis similarly to well-watered plants (Harb et al., 2010). Moreover, negative regulation of jasmonic acid biosynthesis during the later stages of continuous water stress could be beneficial to minimize the negative effects of this hormone on plant growth (Ellis et al., 2002; Harb et al., 2010). Thus, despite smaller plant size, their reproductive ability may be maintained in a stressful environment (Chaves et al., 2002).

Several studies have highlighted the importance of the recovery period following drought for plant resilience (Yaqoobbr et al., 2013; Yousfi et al., 2015; Chen et al., 2016; Sun et al., 2016), and notably for pea plants (Prudent et al., 2016; Couchoud et al., 2020). This period enables mitigation of the negative impact of water stress on ecophysiological processes related to plant nutrition. However, the efficiency of recovery highly depends on plant genotypic strategies for restoring nutrition. For instance, in pea, the genotype Kayanne (that is investigated in the present study) was shown to totally recover, through the fine tuning of its nitrogen nutrition status and yield after a drought event during its vegetative growth period, unlike the genotype Puget, for which a drop in yield was observed (Couchoud et al., 2020).

Drought events can be recurrent and a recovery period can be followed by a new period of water stress. In such a case, plant “memory” from the previous stress event has been pointed out as another mechanism, part of resilience-associated processes (Trewavas, 2003; Bruce et al., 2007; Jacques et al., 2021). Plants memorizing their first period of water stress can modify their response to subsequent stress, during which they can display a faster and/or stronger response. The first stress period (priming) induces various molecular and physiological responses that generate a stress imprint (Hilker and Schmülling, 2019). These impacts of recurrent water stresses on different physiological variables linked to overall growth and yield, on physiological processes such as photosynthetic activity, transpiration, osmotic regulation, and antioxidant system, have been studied in several crop species, including wheat (Wang et al., 2014; Abid et al., 2016; Abid et al., 2017; Abid et al., 2018), corn (Virlouvet et al., 2018), rice (Auler et al., 2017; Aihemaiti et al., 2019; Auler et al., 2021), sugarcane (Marcos et al., 2018), sugarbeet (Leufen et al., 2016), and potato (Ramírez et al., 2015), but not in legumes. Priming enables the maintenance of growth rate through greater leaf water potential, leaf water status, and photosynthetic efficiency, and antioxidant system efficiency, whereas non-primed plants are less likely to maintain their growth rate (Abid et al., 2016; Abid et al., 2018; Auler et al., 2021). However, stress memory has been studied mainly at the molecular level, and particularly in leaves; it is also essential to understand if and how memory of water stress takes place in roots for potentially enhancing plant hydro mineral nutrition.

To our knowledge, studies dealing with pea responses to water stress mainly concern transient stresses; plant memory and acclimation to water stress have rarely been studied in this species. We can take into consideration a study highlighting the impact of recurrent stress on the pea shoot metabolome (Lahuta et al., 2022). Taking into consideration what has been described in the literature for several species, we can hypothesize that pea memory and acclimation to water stress involve different physiological changes in plant adaptation. To test this hypothesis, several ecophysiological variables reflecting water and carbon assimilation and allocation have been either measured or calculated and integrated in an ecophysiological framework, enriched by an analysis of the ionome. Altogether these closely connected variables allow us to decipher and distinguish underlying processes involved in (i) plant response to transient water stress, (ii) stress memory during recurrent water stresses, and (iii) stress acclimation during continuous water stress.

## 2 Materials and methods

### 2.1 Plant growth conditions

**Figure 1:**
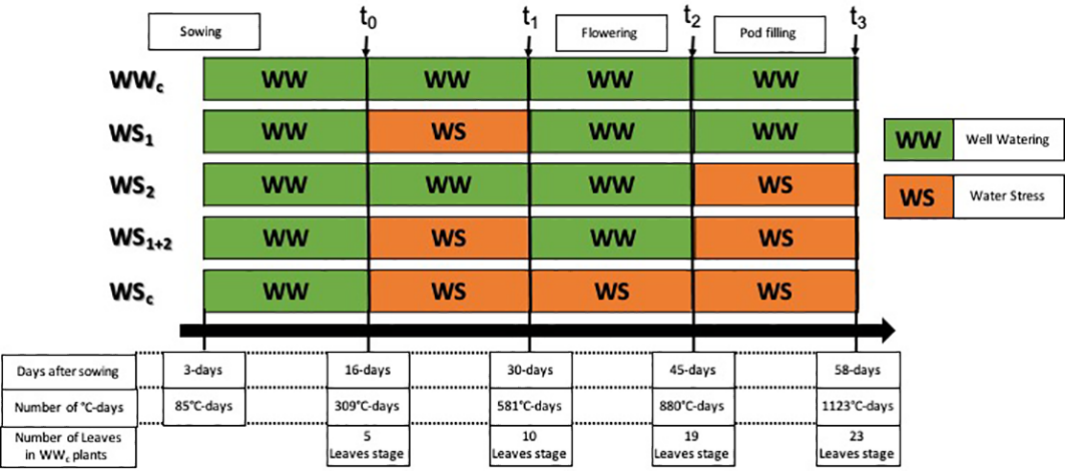
Experimental design used to characterize the response of pea plants to four types of water stress. WS_1,_ transient at vegetative stage; WS_2,_ transient at the reproductive stage; WS_1+2_, recurrent stage; WS_c_, continuous stress. The control treatment corresponds to WW_c_. Well-watered (WW) corresponds to a well-watered period and WS corresponds to a period of water deficit.

Pea seeds (*Pisum sativum* L. cv. Kayanne, obtained from KWS Momont, Mons-en-Pévèle, France) were calibrated, their surfaces sterilized by exposure to 70% ethanol for 1 minute followed by 0.6% sodium hypochlorite for 10 minutes, and then imbibed in distilled water for 2 hours. Seeds were then pre-germinated in germination boxes for 3 days in the dark at 22°C in a Fitoclima S600 germinator (Aralab, Rio de Mouro, Portugal). Seedlings were then transferred to the 4PMI high-throughput phenotyping platform (Dijon, France) in two litters pots filled with a mix of sand and soil harvested in Aiserey, France (**Figure 1**). Soil characteristics are given in **Supplementary Table S1**. Greenhouse environmental conditions were 21.0 ± 1.5°C during the day and 16.6 ± 1.0°C at night, with a photoperiod of 16 h. Supplementary artificial lighting was supplied with sodium lamps (MACS 400W; Mazda, Dijon, France), allowing a mean incident photosynthetically active radiation (PAR) of 280 μE.m^2^.s^–1^. Plants were automatically watered three times per day with N_7_-free nutrient solution [see Voisin et al. (2003) for the composition].

### 2.2 Experimental design and measurements

During the first 2 weeks of growth, plants were watered to reach 90% of substrate water-holding capacity, corresponding to well-watered (WW) conditions. To determine the quantity of the nutrient solution to deliver at each watering, each pot was weighed, and the solution was added to compensate the weight lost due to evapotranspiration. After 2 weeks (t_0_, **Figure 1**), pots were randomly divided into five groups corresponding to the five watering treatments. The first group hereafter called “WW_c_” corresponded to plants that were well watered throughout the experiment. The group called “WS_1_” corresponded to plants that encountered a short period of water stress (2 weeks) during their vegetative stage (transient vegetative stress), the group called “WS_2_” corresponded to plants that encountered a short period of water stress (2 weeks) during their reproductive stage (transient reproductive stress), the group called “WS_1+2_” corresponded to plant that encountered two recurrent water stresses spaced by a re-watering period, and the group called “WS_c_” corresponded to plants that encountered a period of continuous water stress (6 weeks).

After the first 2 weeks of growth (t_0_, **Figure 1**), the watering of WS_c_, WS_1_, and WS_1+2_ plants were stopped to reach 40% of substrate water-holding capacity and was maintained at this level until the end of the water stress period (t_1_, **Figure 1**). After their first water stress period, WS_1_ and WS_1+2_ plants were re-watered for 2 weeks to reach 90% of substrate water-holding capacity (WW), whereas WS_c_ plants watering was maintained at 40% of substrate water-holding capacity for 2 more weeks (t_2_, **Figure 1**). After recovery (for WS_1+2_ plants) or water stress (for WSc plants), WS_c_ plant watering was maintained at 40% of substrate water-holding capacity, whereas for WS_2_ and WS_1+2_ plants, watering was stopped to reach 40% of substrate water-holding capacity for 2 weeks (t_3_, **Figure 1**).

For each treatment (WW_c_, WS_1_, WS_2_, WS_1+2_, WS_c_) five plants were harvested at t_0_, t_1_, t_2_, and t_3_. Before each harvest, stomatal conductance (expressed in mol.m^–2^.s^–1^) was measured on the last fully opened stipule using a porometer (AP4 porometer; Delta T device, Cambridge, United Kingdom). Harvested plants were divided into four samples corresponding to leaves (including stipules and tendrils), stem, roots, and nodules. Leaf and stem areas (Leaves_A_ and Stem_A_, in cm²) were measured using an area meter (LI 3100c area meter; LI-COR, Inc., Lincoln, NE, USA). The root system was thoroughly washed with demineralized water to ensure that the entire root system was free from any soil particles. The root system morphometry was characterized by measuring the taproot length (Taproot_LEN_, in cm), the primary lateral root number (Lateral root_Nb_), the number of nodules localized either on the taproot (Taproot Nodule_Nb_) or on lateral roots (Lateral root Nodule_Nb_). Following each harvest, tissue samples (leaves, stem, roots, and nodules) were dried at 80°C for 48 h. Each organ was weighed to obtain the dry weight of the leaves (Leaves_DW_), stem (Stem_DW_), roots (Root_DW_), and nodules (Nodule_DW_) prior to elemental analyses. Throughout the experiment, each pot was weighed before and after each watering; this allowed us to calculate the plant water uptake (W_Qty_, in g).

### 2.3 Elemental analysis of plant tissues

Each sample was ground to fine powder using MM400 vibratory mixer mill (Retch, France). For each sample, carbon (C) and nitrogen (N) concentrations were measured from 5 mg of ground tissue using the Dumas procedure (Thermo Electron NC 2500 Elemental Analyzer, Courtaboeuf, France).

The other element concentrations (S, P, K, Ca, Mg, Cu, Ni, Mn, B, Zn, Fe, V, Co, Na, and Mo) were measured with a high-resolution, inductively coupled plasma mass spectrometer (HR ICP-MS, Element2, Thermo Fisher) using the PLATIN’ Platform (Caen, France), following methods previously described in Lurthy et al. (2020). For this, a sample of 40 mg of dry powder was submitted to acid digestion composed of 1 mL of nitric acid (HNO_3_), 250 µL of hydrogen peroxide (H_2_O_2_), 900 µL of ultrapure water and 10 µL of internal standard solution of gallium and rhodium before to be diluted to 50 mL with ultrapure water. Then, the solution obtain was filtered with a 0.45 µm Teflon filter. Finally, quantification was obtained by correction of calibration curves by subtracting blank and using internal standards [gallium (Ga) and rhodium Rh)], and evaluated by certified reference plant material (Citrus leaves, CRM NCS ZC73018, Skylab, Metz, France).

### 2.4 Calculated variables and statistical analyses

From measured variables, several other variables were calculated to build an ecophysiological framework originally linking C and N in the plant (adapted from Couchoud et al., 2020) and to calculate water and element uptake and use efficiencies.

The shoot dry weight (*Shoot_DW_*; *expressed in g*) was calculated as:


$$Shoot_{DW}=\;Leaves_{DW\;}+\;Stem_{DW}$$

Where *Leaves_DW_* is the dry weight of leaves (in g) and *Stem_DW_* is the dry weight of stems (in g).

The root system dry weight (*RootSystem_DW_*; *expressed in g*) was calculated as:


$$RootSystem_{DW}=\;Root_{DW}+\;Nodule_{DW}$$

Where *Root_DW_* is the dry weight of root (in g) and *Nodule_DW_* is the dry weight of nodule (in g).

The plant dry weight (*Plant_DW_*; *expressed in g*) was calculated as:


$$Plant_{DW}=\;Shoot_{DW}+RootSystem_{DW}$$

The ratio of shoot dry weight (DW) over root system (root and nodule) DW (*R_Shoot:RootSystem_*) was calculated as:


$$R_{Shoot:RootSystem\;}=\;\frac{Shoot_{DW}}{RootSystem_{DW}}$$

The ratio of nodule dry weight over root dry weight (*R_Nodule_*) was calculated as:


$$R_{Nodule}=\;\frac{Nodule_{DW}}{Root_{DW}}$$

Where *Nodule_DW_* is the dry weight of nodules (in g).

The element quantity in different organs (leaves, stem, root, and nodule) (*Organ*_*Element*_*Qty*__ ; expressed in g) was calculated as:


$$Organ_{Element_{Qty}}=\;Organ_{DW\;}\times Organ_{\left[Element\right]}$$

Where *Organ*_[_ *_Element_*_]_ is the element concentration in organ (in g/g*Organ_DW_*).

And the element quantity in plants (*Plant*_*Element*_*Qty*__ ; expressed in g) was calculated as:


$$Plant_{Element_{Qty}}=\;Leaves_{Element_{Qty}}+\;Stem_{Element_{Qty}}+\;Root_{Element_{Qty}}+Nodule_{Element_{Qty}}$$

That enabled us to calculate the element concentration in plant components (leaves, stem, root, and nodule) (*Plant*_[_ *_Element_*_]_; expressed in g/g*Plant_DW_*) as follows:


$$Plant_{\left[Element\right]}=\;\frac{Plant_{Element_{Qty}}}{Plant_{DW\;}}$$

The relative variation of each variable between time t*_i_* and time t*_i+1_* (Δ*Var_ti_*_→_ *_ti_*_+1_ such as the dry weight, *Element_Qty_* (quantity of element accumulated in plant, expressed in g) or *W_Qty_* (water quantity uptake by root, expressed in g) between two harvests was calculated as:


$$\Delta Var_{ti\to ti+1}=\;\frac{Var_{ti+1}-Var_{ti}}{Var_{ti}}\times 100$$

Where *Var_ti_* is the variable value at the harvest time t*_i_* and *Var_ti_*_+1_ the variable value at the following harvest time t*_i+1_*.

The specific leaf area (SLA; expressed in cm²/g) between two harvests (t_i_ and t_i+1_) was calculated as:


$$SLA=\;\frac{Leaves_{A\;t_{i+1}}-Leaves_{A\;t_{i}}}{Leaves_{DW\;t_{i+1}}-\;Leaves_{DW\;t_{i}}}$$

Where *Leaves_DW_* is the dry weight of leaves (in g) and *Leave_A_* is the projected area of leaves (in cm²).

The radiation use efficiency (RUE, expressed in g/cm²) between two harvests (t_i_ and t_i+1_) was calculated as:


$$RUE=\;\frac{Plant_{DW\;t_{i+1}}-\;Plant_{DW\;t_{i}}}{\int_{ti}^{ti+1}Leaves_{A\;t_{i+1}}.dt}$$

The water use efficiency (WUE; expressed in g/m^3^) between two harvests (t_i_ and t_i+1_) was calculated as:


$$WUE=\;\frac{Plant_{DW\;t_{i+1}}-\;Plant_{DW\;t_{i}}}{W_{Qty\;t_{i+1}}-\;W_{Qty\;t_{i}}}$$

The element use efficiency (EUE; expressed in g of *Plant_DW_*/g) was calculated as:


$$EUE=\;\frac{Plant_{DW\;t_{i+1}}-Plant_{DW\;t_{i}}}{Element_{Qty\;t_{i+1}}-\;Element_{Qty\;t_{i}\;}}$$

The element uptake efficiency (EUpE; expressed g/g of *Root System_DW_*) between two harvests (t_i_ and t_i+1_) was calculated as:


$$EUpE=\;\frac{Element_{Qty\;t_{i+1}}-\;Element_{Qty\;t_{i}}}{\;\int_{ti}^{ti+1}RootSystem_{DW\;}.dt}$$

Statistical analyses were performed with R software (https://www.r-project.org/, v.3.5.2). At each harvest (t_1_, t_2_, t_3_), multiple comparisons were performed between treatments (WS_2_, WS_1+2_, WS_c_, and WW_c_). Kruskal–Wallis tests and Wilcoxon tests were performed *via* Kruskal.test and wilcox.test functions, respectively, and results were displayed *via* ggboxplot function (package ggpubr). Effects were considered significant when the *p*-value was lower than 0.05.

## 3 Results

All primary data are given in **Supplementary Tables S2-S4**, and pictures of plants are given in **Supplementary Figure S5**.

### 3.1 Only single stress period improves water use efficiency

**Figure 2:**
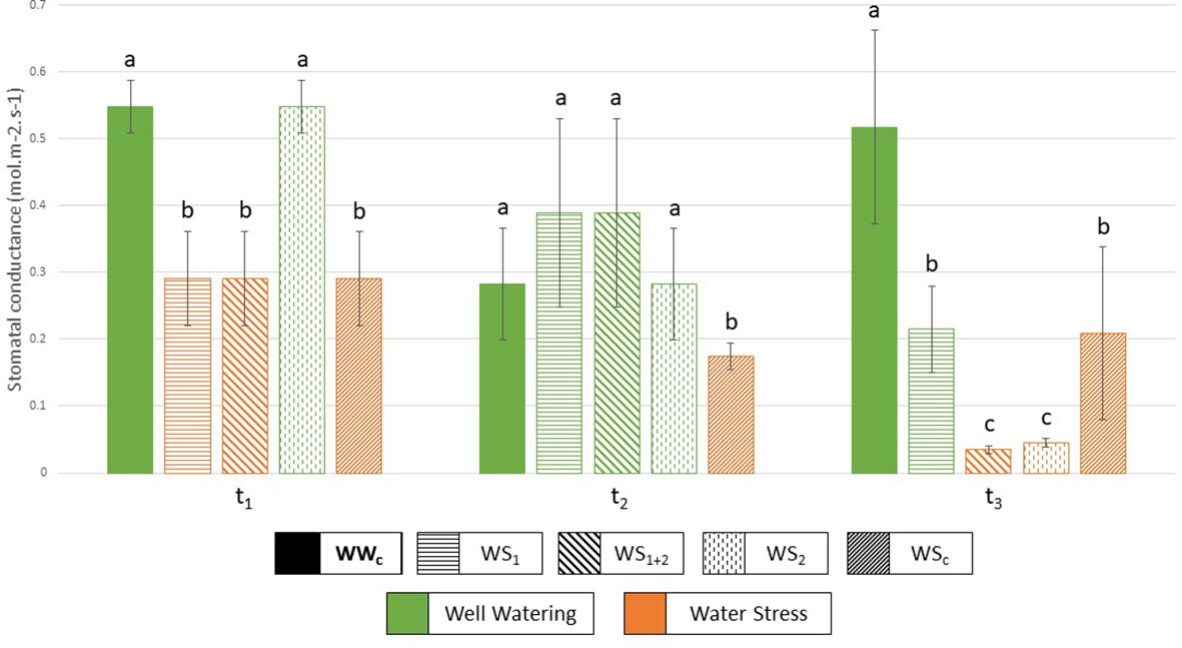
Stomatal conductance (mol.m^–2^.s^–1^) measured at each harvest (t_1_, t_2_, t_3_), and for each treatment. WW_c,_ well-watered control plants; WS_1_, transient vegetative stress; WS_1+2_, recurrent stress, WS_2_, transient reproductive stress, and WS_c_, continuous stress. Green represents a well-watered period and orange, a water-stressed period. Values correspond to means ± SD (*n* = 4 or *n* = 5), letters regroup treatment with a non-significant difference for each harvest.

**Figure 3:**
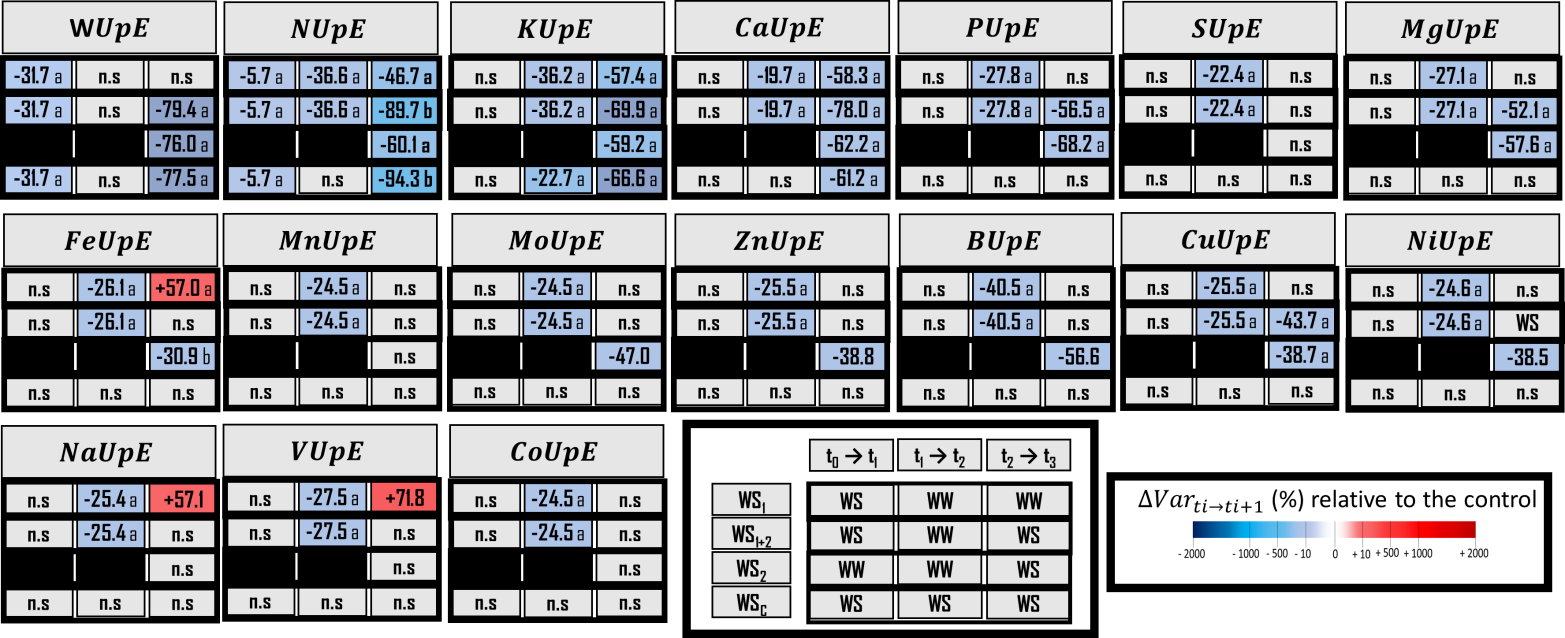
Water (WUpE) and element uptake efficiency (EUpE) for macronutrients, micronutrients and three beneficial elements. Elements (E) characterized are N, nitrogen; K, potassium; Ca, calcium; P, Phosphorus; S, Sulfur; Mg, Magnesium; Fe, Iron; Mn, Manganese; Mo, Molybdenum; Zn, Zinc; B, Boron; Cu, Copper; Ni, Nickel; Na, Sodium; V, Vanadium, and Co, Cobalt. The effect of each treatment (WS_1_, transient vegetative stress; WS_1+2_, recurrent stress; WS_2_, transient reproductive stress; WS_c,_ continuous stress) was expressed relatively to control plants (WW_c_) at each harvest (t_1_, t_2_, t_3_) (ΔVar t_i_→t_i+1_). Boxes were colored in red when the treatment induced an increase of the variable, and in blue when the treatment induced a decrease of the variable compared with conrol plants (WW_c_); the intensity of the effect was characterized by the scale of color and the value (percent compared with WW_c_) present in the box. Data are presented as a percentage relative to control plants (*n* = 4 or *n* = 5). Primary data are available in **Supplementary Table S4**. Letters regroup treatments with non-significant difference for each harvest.

**Figure 4:**
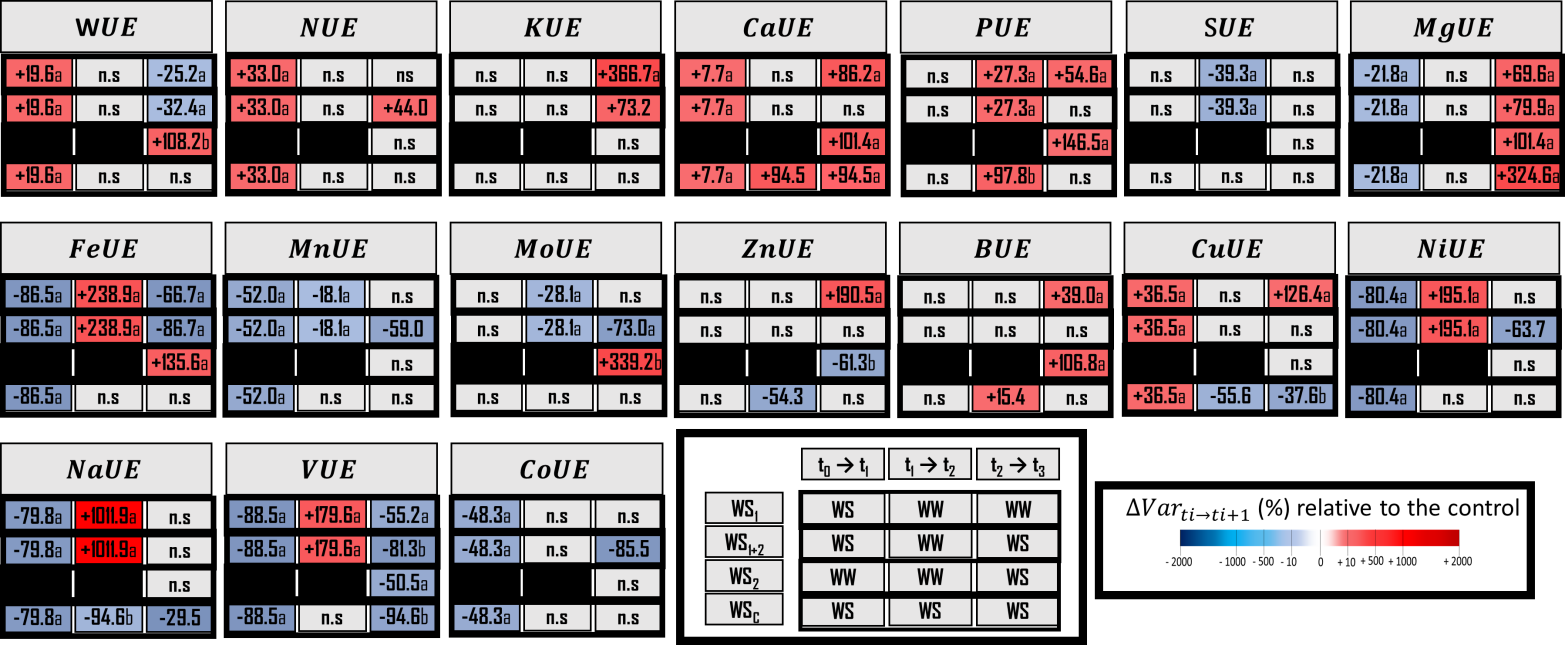
Water use efficiency (WUE) and element use efficiency (EUE) for macronutrients, micronutrients and three beneficial elements. Elements (E) characterized are N, Nitrogen; K, Potassium; Ca, Calcium; P, phosphorus; S, sulfur; Mg, magnesium; Fe, iron; Mn, manganese; Mo, molybdenum; Zn, zinc; B, boron; Cu, copper; Ni, nickel; Na, sodium; V, vanadium, and Co, cobalt. The effect of each treatment (WS_1_, transient vegetative stress; WS_1+2_, recurrent stress; WS_2_, transient reproductive stress; WS_c_, continuous stress) was expressed relatively to control plants (WW_c_) at each harvest (t_1_, t_2_, t_3_; *n* = 4 or *n* = 5) (ΔVar t_i_→t_i+1_). Boxes were colored in red when the treatment induced an increase of the variable, and in blue when the treatment induced a decrease of the variable compared with conrol plants (WW_c_); the intensity of the effect was characterized by the scale of color and the value (percent compared with WW_c_) present in the box. Primary data are available in **Supplementary Table S4**. Letters regroup treatment with non-significant difference for each harvest.

To characterize plant water management, several traits were measured and calculated for each water stress treatment (WS_1_, WS_2_, WS_1+2_ and WS_c_) such as stomatal conductance (**Figure 2**), water uptake, and use efficiencies (**Figures 3**, **4**). Under recurrent stress during the first stress period (WS_1+2_ at t_1_), water uptake efficiency (WUpE) and stomatal conductance were decreased respectively by 22.8% and 31.7% (**Figures 3**, **2**), whereas water use efficiency (WUE) was increased by 95.7% (**Figure 4**). However, during the recovery period (WS_1+2_ at t_2_) all these variables returned to the values of control plants. Then during the second stress period (WS_1+2_ at t_3_), stomatal conductance and WUE decreased. The impact on stomatal conductance was equivalent under transient reproductive stress (WS_2_ at t_3_), under which WUpE was decreased by 76.0%. Whereas, WUE decrease was equivalent for recurrent (WS_1+2_) and single stress at vegetative stage (WS_1_) during reproductive period (t_3_). And under continuous stress of 6 weeks (WS_c_ at t_3_), stomatal conductance was less decreased by 54.3% and WUpE was decreased by 77.5%.

### 3.2 Two strategies for soil prospection: Increased taproot growth under WS_2_ and enhanced lateral root initiation under WS_c_

**Figure 5:**
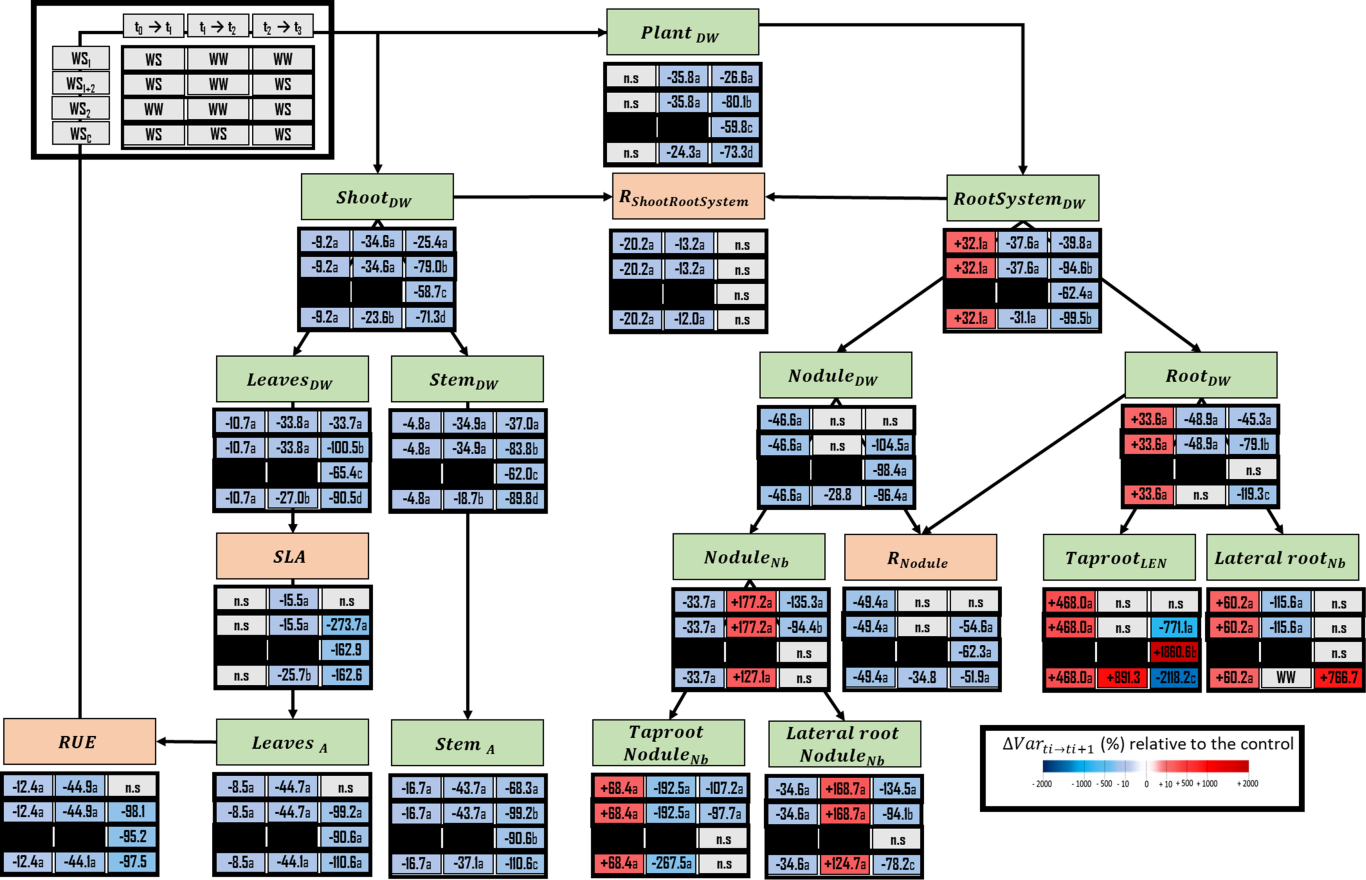
Ecophysiological framework highlighting several variables related to carbon fixation or allocation within plant, that have been either measured or calculated. Lateral root_Nb_, lateral root number; Lateral root Nodule_Nb_, number of nodules located on the lateral roots; Leaves_DW_, leaves dry weight; Leaves_A_, Leaves projected area; Nodule_DW_, nodule dry weight; Nodule_Nb_, total nodule number; Plant_DW_, plant dry weight; R_Nodule_, ratio of nodule dry weight over the root dry weight; R_Shoot_ : _RootSystem_, ratio of shoot dry weight over the whole root system dry weight; Root_DW_, root dry weight; RootSystem_DW_, root System (root and nodule) dry weight; RUE, radiation use efficiency; Shoot_DW_, shoot dry weight; Stem_A_, stem projected area; Stem_DW_, stem dry weight; Taproot_LEN_, taproot length; Taproot Nodule_Nb_, number of nodules located on the taproot; SLA, specific leaf area. The effect of each treatment (WS_1_, transient vegetative stress; WS_1+2_, recurrent stress; WS_2_, transient reproductive stress; WS_c_, continuous stress) was expressed relatively to control plants (WW_c_) at each harvest (t_1_, t_2_, t_3;_ n=4 or 5) (ΔVar t_i_→t_i+1_). Boxes were colored in red when the treatment induced an increase of the variable, and in blue when the treatment induced a decrease of the variable; the intensity of the effect was characterized by the scale of color and the value (percent compared with WW_c_) present in the box. Primary data are available in **Supplementary Table S2**. Letters regroup treatment with non-significant difference for each harvest.

The first water stress period at the vegetative stage (t_1_) induced numerous changes related to plant morphology and carbon fluxes (**Figure 5**). First, carbon was preferentially allocated to the nodulated root system, at the expense of shoots. Indeed, the shoot dry weight accumulation (including both leaves and stem) of water-stressed plants was reduced by 9.25% when compared with well-watered plants, because of a decrease in leaf area of 8.49% and a decrease in radiation use efficiency (RUE) of 12.4%. At the same time, the dry weight of the root system increased by 32.1% because of the increase in taproot dry weight (+68.4%), the length of which also increased, and of the lateral roots’ dry weight (+60.2%), which were were numerous in water-stressed plants than in well-watered plants. Moreover, the ratio of nodule dry weight to root dry weight (R_Nodule_) was decreased (by 49.4%), indicating during this stress period a higher allocation of C to the root than to nodule. This explained the decrease of nodule dry weight accumulation and nodule initiation, respectively, by 46.6% and 33.6%. During the following recovery period (WS_1+2_ at t_2_) the variables which are related to the photosynthetic C fixation and to the allocation of C in the shoot were almost unchanged, except for the specific leaf area (SLA), which decreased by 15.5% (**Figure 5**). On the contrary, opposite responses were observed between the end of the first water stress period and the end of the recovery period in below-ground parts. Indeed, root system DW accumulation was decreased, and nodule initiation increased, respectively by 37.6% and 177.2%.

During the reproductive stage (t_3_), whatever type of water stress was considered (transient, WS_1_, or WS_2_; recurrent, WS_1+2_, or continuous WS_c_), we found that most of the traits reflecting photosynthetic carbon fixation and allocation were similarly impacted. However, the intensity of this impact depended on the type of stress. For instance, plant dry weight accumulation was more negatively impacted under recurrent stress (–80.1%) than under continuous stress (–73.3%), single reproductive stress (–59.8%), and single vegetative stress (–26.6%). On the other hand, traits reflecting root morphology were typically affected by each of the four water stress treatments. When compared with well-watered plants, taproot elongation was increased under transient reproductive stress (+1860%) but decreased under recurrent stress (–771.0%) and even more under continuous stress (–2118.2%). The number of lateral roots initiated during this period was not affected by transient reproductive stress or a recurrent stress but was greatly decreased (+766.7%) under continuous stress. Conversely, the number of nodules initiated during this period was decreased by 94.4% under recurrent stress and by 135.3% transient vegetative stress at reproductive period.

### 3.3 Element uptake efficiencies were more negatively impacted under continuous water stress and element use efficiencies were more negatively impacted under recurrent water stress

To characterize pea mineral nutrition during the four types of water stress [transient stress (vegetative, WS_1_; or reproductive, WS_2_), recurrent stress (WS_1+2_), and continuous stress (WS_c_)], an analysis of the plant ionome was performed in the different organs of the plant and allowed to characterize both the uptake efficiency and the use efficiency of 16 nutrients and beneficial elements.

When water stress occurred during the vegetative stage (WS_1+2_ at t_1_), 11 out of the 16 elements were impacted for their uptake efficiencies (UpE) or for their use efficiencies (UE), or for both (**Figures 3** and **4**). However, only N uptake efficiency (NUpE) and use efficiency (NUE) and use efficiencies responded in a compensatory way, with a lower level of uptake efficiency (–5.7%) that could be compensated for by a higher use efficiency of this element (+32.7%). The use efficiencies of Ca and Cu were increased (by 7.7% and 36.5%, respectively). Conversely, Mg, Fe, Mn, Ni, Na, V and Co use efficiencies were decreased (by 21.8%, 86.5%, 52.0%, 80.4%, 79.8%, 88.5%, and 48.28%, respectively). Under water stress at the vegetative stage, VUE (Vanadium Use Efficiency, -88.5%) and FeUE (Iron Use Efficiency, -86.5%) were the most negatively impacted. Interestingly, K, P, S, Mo and Zn and use efficiencies were not impacted by the water stress at vegetative stage. Similarly, to what was previously observed for variables related to root morphology, it appeared that for most of the elements (except for NUpE, KUE, ZnUE, and BUE) their uptake or use efficiencies were drastically changed during the recovery period following the water stress (WS_1+2_ at t_2_). Moreover, other elements were impacted by their uptake efficiencies (KupE, +22.3%; CaUpE, –19.7%; PUpE, –27.8%; SUpE, +81.0%; MgUpE, –24.5%; FeUpE, –26.1%; MnUpE, –24.5%; MoUpE, +80.9%; ZnUpZ, –25.5%; BUpE, –40.5%; CuUpE, –25.5%; NiUpE, –24.5%; NaUpE, –25.4%; VUpE, –27.5%; and CoUpE, –24.5%) or their use efficiencies (PUE, +27.3%; and SUE, +39.3%). During recovery, MnUE decreased by a smaller amount (by 18.4%) than in the first stress period (WS_1+2_ at t_2_).

During the reproductive stage (t_3_), either uptake efficiencies, use efficiencies, or both were impacted. In terms of elements and the three types of stress, MgUpE was decreased and MgUE was increased, by –52.5% and +79.9%, respectively; for recurrent stress, by –57.9% and +181.9%, respectively, for transient stress; and only MgUE was increased, by +323.6%, for continuous stress. Moreover, CaUpE (Calcium Uptake Efficiency) and CaUE (Calcium Use Efficiency) were similarly regulated under transient vegetative or reproductive stress (–58.3% and +86.2%; –62.2% and +101.4%, respectively) and continuous stress (by –61.2% and +94.5%). In terms of transient reproductive stress (WS_2_), the most negatively impacted uptake efficiency was PUpE (–68.2%) and the only use efficiency negatively impacted was VUE (–50.2%). In terms of recurrent stress, antagonistic regulations were observed, such as the decrease of NUpE (–86.7%) and the increase of NUE (+44.1%). NUpE was the uptake efficiency that was most decreased (–86.7%) and the FeUE use efficiency was most decreased (–86.7%). Moreover, NUpE and VUE were less impacted under transient vegetative stress (WS_1_ at t_3_) (respectively, –46.7% and –55.2%) than under recurrent stress (WS_1+2_ at t_3_) (respectively, –86.7% and –81.4%). Whereas, these two types of stress impact KUpE, MgUE, and FeUE in a similar way. Under continuous stress, CuUE (–55.6%) and NaUE (–94.62%) were the only element use efficiencies that were decreased as soon as after 4 weeks of stress. However, the uptake efficiencies of K were negatively impacted after 4 weeks of stress. After 6 weeks of stress, three uptake efficiencies were negatively impacted (NUpE, –94.3%; KUpE, –66.6%; CaUpE, –61.2%) Finally, under continuous stress, NaUE had the most decreased use efficiency (–94.6%).

### 3.4 Water stress modulated plant element concentrations progressively from roots to shoot

**Figure 6:**
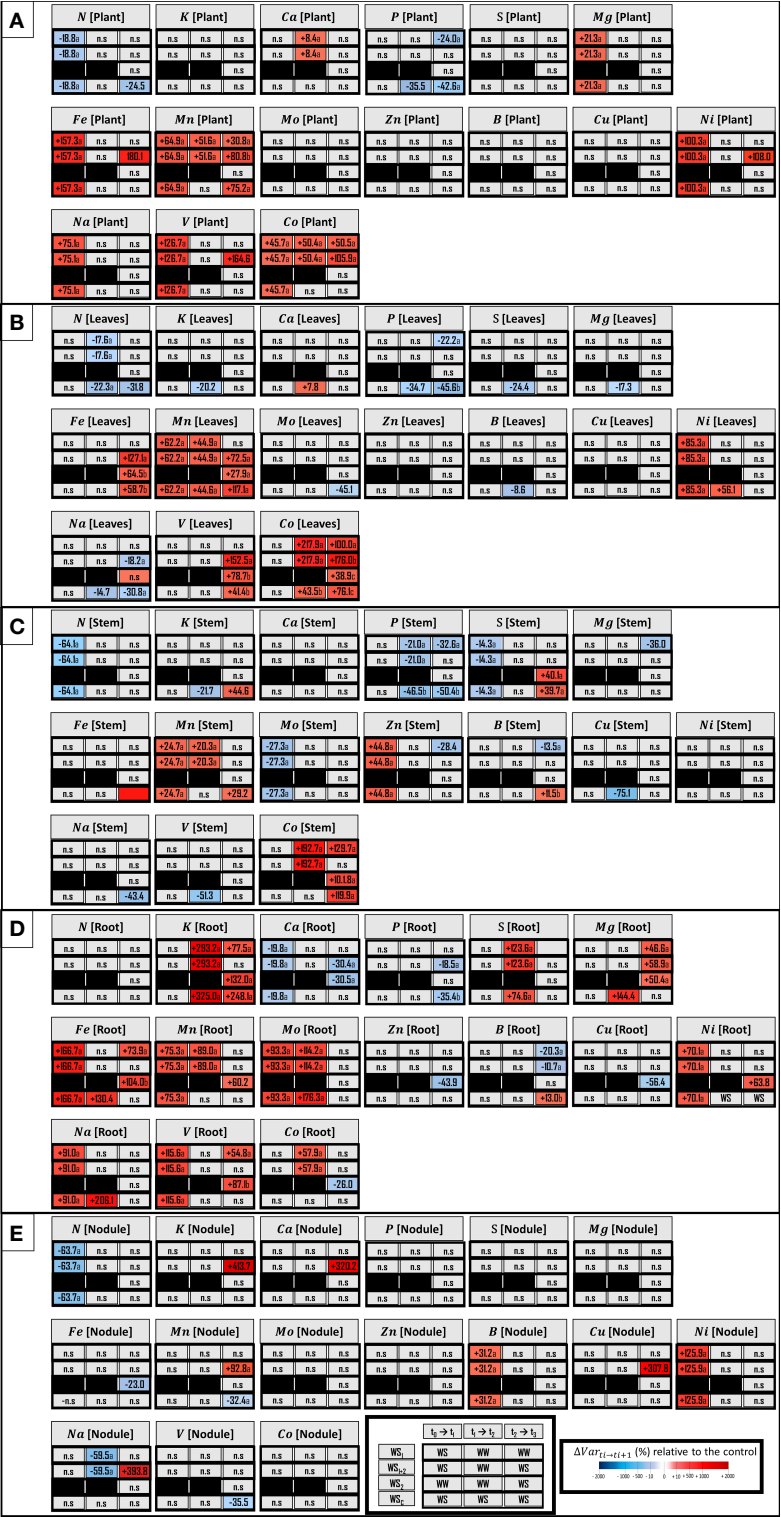
Concentrations of macro-nutrients, micro-nutrients and three beneficial elements in whole plant **(A)** and leaves **(B)**, stem **(C)**, roots **(D)**, nodules **(E)**. Elements (E) characterized are N, nitrogen; K, potassium; Ca, calcium; P, phosphorus; S, sulfur; Mg, magnesium; Fe, iron; Mn, manganese; Mo, molybdenum; Zn, zinc; B, boron; Cu, copper; Ni, nickel; Na, sodium; V, vanadium, and Co, cobalt. The effect of each treatment (WS_1_, transient vegetative stress; WS_1+2_, recurrent stresses; WS_2_, transient reproductive stress; WS_c_, continuous stress) was expressed relatively to control plants (WW_c_) at each harvest (t_1_, t_2_, t_3;_ n=4 or 5) (ΔVar t_i_→t_i+1_). Boxes were colored in red when the treatment induced an increase of the variable, and in blue when the treatment induced a decrease of the variable; the intensity of the effect was characterized by the scale of color and the value (percent compared with WW_c_) present in the box. Primary data are available in **Supplementary Table S3**. Letters regroup treatment with non-significant difference for each harvest.

A description of the plant ionome based on the element concentrations of each organ was performed for each water stress treatments (**Figure 6**). After the first stress period (WS_1+2_ at t_1_), eight element concentrations were modulated: seven were increased (C, Mg, Fe, Mn, Ni, Na, V and Co) among which the Fe concentration was most improved (+157.3%), and the N concentration was the only negatively altered concentration (-18.8%) (**Figure 6A**). Moreover, roots were the organ most impacted by the six elements with increased concentrations (Fe, Mn, Mo, Ni, Na and V) among which Fe concentration (+166.6%) was the most increased, and Ca concentration was the only decreased concentration (–19.8%) (**Figure 6D**). Leaves were the organ least impacted by stress, with only two element concentrations that were increased, among which the Ni concentration (+85.3%) was most increased (**Figure 6B**). Moreover, in leaves, the Zn concentration was most increased (+44.7%) and the N concentration was most decreased (–64.1%). In nodules, the Ni concentration was also greatly increased (+125.9%) and the N concentration was similarly decreased (–63.7%) (**Figure 6E**).

During the recovery period after the first water stress period (WS_1+2_ at t_2_), the Mn and Co concentrations continued to increase at the whole plant level (**Figure 6A**). Roots were still the most impacted organ during recovery (**Figure 6D**). The concentrations of Mn and Mo were enhanced in this organ, but the K concentration was most amplified (+293.2%). The organs least impacted were the nodules, in which the Na concentration was the only decreased concentration (–59.4%) (**Figure 6E**).

During the second stress period (WS_1+2_ at t_3_), Mn and Co concentrations remained increased at the whole plant level, and the Fe concentration was most increased (+180.1%) (**Figure 6A**). However, although the Co concentration was equivalently impacted under transient vegetative stress (WS_1_ at t_3_), the Mn concentration was less impacted. Moreover, in root Mg and B concentrations were equivalently impacted by these two types of stresses (**Figure 6D**). Under recurrent stress, stem was the organ that was not affected while nodules were the most impacted (five element concentrations were enhanced). In nodules, the K concentration was the most increased (+413.7%) (**Figure 6E**).

After 4 weeks of continuous stress (WS_c_ at t_2_), the P concentration was the only concentration that was decreased at the whole plant level (–35.5%) (**Figure 6A**). The leaves were the compartment most impacted by the four elements with increased concentrations (Ca, Mn, Ni and Co) and the seven elements with decreased concentrations (N, K, P, S, Mn, B and Na) (**Figure 6B**). In leaves, the element with the most increased concentration was Ni (+56.1%) while the element with the most reduced concentration was P (–34.7%). The stem was the least impacted organ, with only four elements whose concentrations were decreased (K, P, Cu and V) (**Figure 6C**). Then, after 6 weeks of continuous stress (WS_c_ at t_3_), the P concentration was still decreased by 42.6% (as was as the N concentration) and Mn was the only element whose concentration increased (+75.2%) at the whole plant level. Moreover, leaves and stem were the most impacted components, with eight element concentrations that increased or decreased. In the stem, six element concentrations increased (K, S, Fe, Mn, B and Co), among which the Fe concentration was most increased (+193.5%) and the P and Na concentrations were most decreased (–50.4% and -43.4%, respectively). Nodules were only slightly affected by decreased Mn and V concentrations (**Figure 6E**).

Under single transient stress at the reproductive stage (WS_2_ at t_3_), no change in element concentration was observed at the whole plant level (**Figure 6A**). Moreover, roots were the organs that were the most impacted by the six elements with increased concentrations (K, Mg, Fe, Mn, Ni and V) among which K concentration was the most increased (+132.0%) and four element concentrations that decreased (Ca, Zn, Cu and Co), among which the Cu concentration was most decreased (–56.3%) (**Figure 6D**). Finally, nodules and aerial parts were the organs least impacted (**Figure 6E**).

## 4 Discussion

In this study, plant strategies related to hydromineral nutrition status maintenance or restoration were characterized according to the different types of water stress: single transient stress at the vegetative stage (WS_1_), single transient reproductive stress (WS_2_), recurrent stress (WS_1+2_), and continuous stress (WS_c_). Thus, plant strategies (hereafter, we will refer to plant responses that may play a role in resilience mechanisms to water stress as “strategies”) that could be involved in resilience to water stress present common and typical response among the different types of stress. Plants were described at the whole-plant level through ecophysiological variables related to water, soil mineral resource uptakes and uses, and, finally, their ionome composition.

### 4.1 The shoot compartment was negatively impacted, whatever type of water stress it encountered

Shoot compartment was negatively impacted whatever the type of stress encountered by the plant and the period in the growth cycle at which the stress occurred (**Figure 5**). However, the decrease of plant growth, and more specifically of its shoot, was greater under recurrent stress and during continuous stress than single reproductive stress. However, the impact on shoot growth of stress at reproductive stage during recurrent stress (WS_1+2_ at t_3_, -79.0%) could represent cumulative effect of single vegetative stress (WS_1_ at t_3_, -25.4%) and single reproductive stress (WS_2_ at t_3_, -58.7). A general trend was observed for all stresses, whatever the period of the plant cycle (except WS_1_ at t_3_). Mn was the only element which concentration was systematically enhanced in leaves (**Figure 6B**). This element is known to be involved in several processes related to photosynthesis (Goussias et al., 2002; Schmidt et al., 2016), ATP synthesis (Pfeffer et al., 1986; Ahanger et al., 2016), RuBP carboxylase reaction (Houtz et al., 1988; Bloom and Lancaster, 2018), and biosynthesis of fatty acid, acyl lipid, and proteins (Ness and Woolhouse, 1980; Millaleo et al., 2010; Tripathi et al., 2015). Moreover, Mn plays a role in antioxidant response under water stress *via* its requirement for superoxide dismutase (Sevilla et al., 1980; Bridges and Salin, 1981; Sandmann and Böger, 1983; Polle et al., 1992). So, even if we have not demonstrated that plants experienced a Mn deficiency during drought, one could suggest that an increased concentration of Mn in leaves could improve carbon fixation and thus plant growth under water stress. Moreover, under continuous stress (WS_c_ at t_3_), the increase of Mn concentration in leaves was amplified when compared with recurrent (WS_1+2_ at t_3_) and transient reproductive stresses (WS_2_ at t_3_). This could explain the smaller reduction of plant and shoot growth under continuous stress as compared with recurrent stress. Moreover, some studies have highlighted the beneficial role of a foliar application of Mn for plants grown under water deficit, as it could improve growth parameters and N_2_ fixation (Purcell et al., 2000; Karim et al., 2012; Ghorbani et al., 2019). Concomitantly, under a continuous stress, the higher stomatal conductance that was observed could be linked to a higher photosynthetic rate than during recurrent or transient stresses. It thus appears that pea plants, by reducing their growth, seem to induce an acclimation process leading to the maintenance of a physiological activity similar to that observed in control plants. This limitation of energy-consuming processes during the first weeks of drought reduced plant growth on the long term (Harb et al., 2010).

As with the concentration of Mn, concentrations of Fe, Co, and V were increased regardless of the type of water stress encountered at the reproductive stage (except for Fe and V, WS_1_ at t_3_), with higher increases observed under recurrent stress (**Figure 6A**). In terms of the Co concentration, its increase under recurrent stress (WS_1+2_ at t_3_, +176.0%) seemed higher than the cumulative impact of single vegetative stress (WS_1_ at t_3_, +100.0%) and single reproductive stress (WS_2_ at t_3_, +38.9%). In the same way as for Mn, increases in the concentrations of Fe and V in leaves could also help the plant to maintain its growth under water stress at the reproductive stage. Indeed, Fe is known to be involved in chlorophyll biosynthesis, photosynthesis, and respiration (Halvin et al., 1999; Rehman et al., 2021), and the concentration of V can enhance plant growth *via* a higher tissue elasticity, thus enabling cell expansion (García-Jiménez et al., 2018).

On the other hand, Na concentration in leaves only increased under transient reproductive stress, but decreased under recurrent and continuous stress at the reproductive stage (**Figure 6A**). Therefore, the increased concentration of Na in leaves could be beneficial *via* its osmoticum role and its growth promotion under the non-limiting conditions of K (Lehr and Wybenga, 1955; Tinker, 1965; Milford et al., 1977; Pessarakli et al., 2015).

Although more focused investigations are needed to assess the different roles of Na, Mn, Fe and V, these elements may be beneficial for various plant biological processes despite a lower plant and shoot growth rate under water stress at the reproductive stage. For instance, fine measures of physiological parameters (such as photosynthesis) under different levels of Na, Mn, Fe and V could validate the implication of their concentration increase on the water stress tolerance process.

### 4.2 Within the root system, priming inhibited nodule initiation and acclimation modulated root architecture

The root system was impacted in a different manner by each of the three types of water stress applied at the reproductive stage. Taproot elongation was promoted only under transient reproductive stress (WS_2_ at t_3_), whereas lateral root initiation increased only under continuous stress (WS_c_ at t_3_). On the other hand, recurrent stress (WS_1+2_ at t_3_) was the only stress applied at the reproductive stage that decreased nodule initiation, with nodulation most typically occurring on the taproot. However, the same trend was observed at this period in plants for which a single period of stress applied at the vegetative stage (WS_1_ at t_3_). Therefore, at the root system scale, different responses were induced under water stress during the reproductive period, depending on the type of stress.

#### 4.2.1 After single reproductive transient stress, plant promoted taproot growth and maintained nodule initiation

The typical impact of single reproductive transient stress was mostly observed in roots whose taproot length was increased and whose nodule numbers remained stable (**Figure 5**). The increase of the conctration of V in roots is also an important element for increased taproot growth rate under single reproductive stress. Indeed, this element has been shown to improve taproot elongation *via* its role in the elasticity of tissues associated with cell expansion (García-Jiménez et al., 2018; Chen et al., 2021).

Moreover, the modification of root and nodule ionomes (**Figures 6D, E**) could in turn impact nodule growth, development, and activity. On one hand, the amount of N fixed by nodules depends on structural components such as nodule number and dry weight. On the other hand, it depends on functional component like N_2_ fixation activity. Several mineral elements were important for both the establishment and the maintenance of these two components. Concerning the structural component of legume symbiosis, Fe impacts nodule formation (Su et al., 2008) and, as Cu is also critical for nodule dry weight accumulation (Hemantaranjan and Garg, 1986; Tang et al., 1990; O’Hara et al., 1993; Tang, 2001; Rotaru and Sinclair, 2009).

The larger concentration of Fe in roots observed in our study could explain the maintenance of nodule initiation during the reproductive stage (**Figure 5**). Similarly, the observed decrease of nodule dry weight accumulation could be linked to both the decrease of Fe concentration in nodules and the decrease of Cu concentration in roots (**Figure 6D**). Regarding the functional component of N_2_ fixation, decreased concentrations of Fe in nodules and Cu in roots could negatively impact nodule activity. Indeed, Fe is an essential element for N_2_ fixation, because of its role in bacteroid and nitrogenase activity (Kaczor et al., 1994; Rotaru and Sinclair, 2009) and Cu deficiency has been shown to negatively impact leghaemoglobin concentration (Snowball et al., 1980; Seliga, 1993; Seliga, 1998). Moreover, increased Ni and V concentrations in roots could enhance nodule activity. Indeed, Ni is essential to nitrogenase activity in many rhizobial bacteria (Albrecht et al., 1979; Brito et al., 1994; Cammack, 1995; Freitas et al., 2019).

Further studies should aim at understanding the most limiting elements for nitrogen fixation in peas among Fe, Cu, and Ni are, and confirm if an increase of these element concentrations in nodule or roots could directly positively impact the structural and functional components of N_2_ fixation under water stress. This could help to identify which elements ought to be preventively supplemented to the crop before the appearance of potential transient water stress.

#### 4.2.2 Priming limited plant growth and promoted element concentrations of tissues

Under recurrent stress, ecophysiological memory was characterized by analogy with memory characterization at the molecular scale (Ding et al., 2013; Ding et al, 2014; Jacques et al., 2021), by quantifying the changes induced by the second stress period regarding those induced by a single stress period (**Supplementary Material S6**). The typical impact of recurrent stress (WS_1+2_ at t_3_) on the root system consisted in the decrease of the number of nodules initiated on both the taproot and the lateral roots during the stress such as observed during the reproductive period after a transient vegetative stress (WS_1_ at t_3_) (**Figure 5**). However, the decrease in the number of nodules initiated on lateral roots was lower under recurrent stress compared with the decrease that occurred after transient vegetative stress. This decrease of nodule initiation during the second stress period could be explained by the previous increase of nodule initiation during the recovery period following the first water stress period. Nodules were the only organs that displayed the most intensive ionome regulation under recurrent stress, as illustrated by increases of K, Ca, Cu and Na concentrations (**Figure 6E**), but were not impacted under transient vegetative stress. In faba bean or yellow lupin, Cu accumulation in nodules was shown to improve nodule dry weight accumulation and leghaemoglobin concentration (Seliga, 1998). Under recurrent water stress, an increase of Cu concentration in nodules may improve water stress tolerance in the pea plant, with a lower negative impact on nodule development and function. It seems consistent with the lower decrease of nodule dry weight accumulation under recurrent stress, regarding transient reproductive stress (**Figure 5**). Finally, although recurrent stress had a more negative impact on plant growth than transient reproductive stress, changes related to the nodule compartment (initiation, growth, elemental concentration) may be considered a ecophysiological imprint of previous water stress. Moreover, this imprint could be more beneficial if a second period of stress occurs. This highlights the importance of the trade-off between memory and the absence of memory if a second period of stress does not occur (Crisp et al., 2016; Jacques et al., 2021).

#### 4.2.3 The acclimation of plants during continuous stress, enhanced plant soil exploration, and delayed nodule initiation

The typical impact of continuous stress on the root system consisted in an increase of lateral root number after 6 weeks of stress (**Figure 5**). This promotion of lateral root initiation could be putatively and partly explained by an increase of B concentration in roots that could also promote root hair formation, N_2_ fixation, and ion uptake (Gupta and Solanki, 2013; Shireen et al., 2018). Because the limit between plant deficiency and excess for B has been reported to be quite low, the small increase in B concentration observed could limit toxic effect of this element (Dell and Huang, 1997). However, contrary to what has been previously observed in the literature, the increase in the concentration of B did not seem to be beneficial for root dry weight or elongation in pea roots (Hodecker et al., 2014; Shireen et al., 2018), whereas changes in root architecture could be beneficial for soil water and nutrient uptake by other plants (Vilches-Barro and Maizel, 2015). It will thus be necessary to determine the importance of B concentration to the root architecture in pea plants submitted to water stress under different B concentrations.

On the other hand, for nodules, a similar pattern to that observed under recurrent stress was highlighted under long-term continuous stress: although the initiation of nodules was promoted after 4 weeks of stress, it declined after 6 weeks. Stress memory and acclimation to water stress both seem to induce a delay of nodulation, as already observed during the recovery period following drought in pea (Couchoud et al., 2020). From a functional point of view, V concentrations were decreased under continuous stress. Yet, V is a beneficial element for nitrogenase activity that could be decreased under continuous stress (Imtiaz et al., 2015; Altaf et al., 2020). Finally, the acclimation of pea plants after 6 weeks of water stress reduced plant growth, with changes in root system architecture leading to extended soil exploration. It could be interesting to characterize the impact of V concentration under water stress on N_2_ fixation, to confirm the potential negative impact of the decrease in the concentration of V on the functional component of nodulation.

## 5 Conclusion

Our results allowed the identification of different plant strategies related to acclimation or stress memory according to the type of water stress encountered by the plant during its growth cycle. The type of stress can be characterized in leaves or roots by ionomic imprint of each stress. The increased concentration of Mn in leaves seems to be a common response of pea plants to water stress, whatever the stage of the plant growth cycle or type of stress encountered. Moreover, under single transient reproductive stress, Na and Mn concentrations in leaves may be involved in the maintenance of shoot growth. On the other hand, the modification of root system seems to be a typical response to considered water stress. During acclimation to long-term water stress, soil prospection was promoted *via* a higher lateral root number that could also be related to high levels of B in this organ. Finally, under recurrent stress, the recovery period between the two stress periods was beneficial for N_2_ fixation through the increase of nodule initiation that could be thus considered as an ecophysiological imprint of a first stress period.

## Data availability statement

The original contributions presented in the study are included in the article/**Supplementary Material**. Further inquiries can be directed to the corresponding author.

## Author contributions

CS, SP, and MP conceived the project. CJ, SG, FL, and MP performed the experiments. CJ analyzed the data. All authors contributed to the article and approved the submitted version.
