# Supplementary material for: Memory or acclimation of water stress in pea rely on root system's plasticity and plant's ionome modulation
Source: Front Plant Sci. 2023 Jan 25;13:1089720. doi: 10.3389/fpls.2022.1089720 (PMC9905705; doi:10.3389/fpls.2022.1089720)
Supplement: Supplementary file 1 [file DataSheet_1.pdf]

Supplementary Materials

Memory or acclimation of water stress in pea rely on root system plasticity and plant ionome modulation.

Cécile Jacques[1], Sylvie Girodet[1], Fanny Leroy[2], Sylvain Pluchon[3] , Christophe Salon[1] and Marion Prudent[1]*

[1] Agroécologie, INRAE, Institut Agro, Univ. Bourgogne, Univ. Bourgogne Franche-Comté, F-210003 Dijon, France

[2] Plateforme PLATIN', US EMerode, Normandie Université, Unicaen, Caen, France5

[3] Laboratoire de Nutrition Végétale, Centre Mondial de l'Innovation Roullier, TIMAC AGRO, Saint Malo, France

*** Correspondence: Marion Prudent, marion.prudent@inrae.fr**

Supplementary Material S5

Images taken throughout the experiment in the 4PMI Platform in Dijon, France.

A) Overview of the greenhouse experiment;

B) Picture of pea plants at the first harvest (309 °C-day, t_0) grown under the five treatments;

C) Picture of pea plants at the second harvest (581 °C-day, t_1) grown under the five treatments;

D) Picture of pea plant at the third harvest (880 °C-day, t_2) grown under the five treatments;

E) Picture of pea plant at the last harvest (1123 °C-day, t_3) grown under the five treatments;

WW_c : well-watering; WS_1 :vegetative transient water stress; WS_2 : reproductive transient water stress; WS_{1+2} : recurrent water stress; WS_c : continuous water stress.

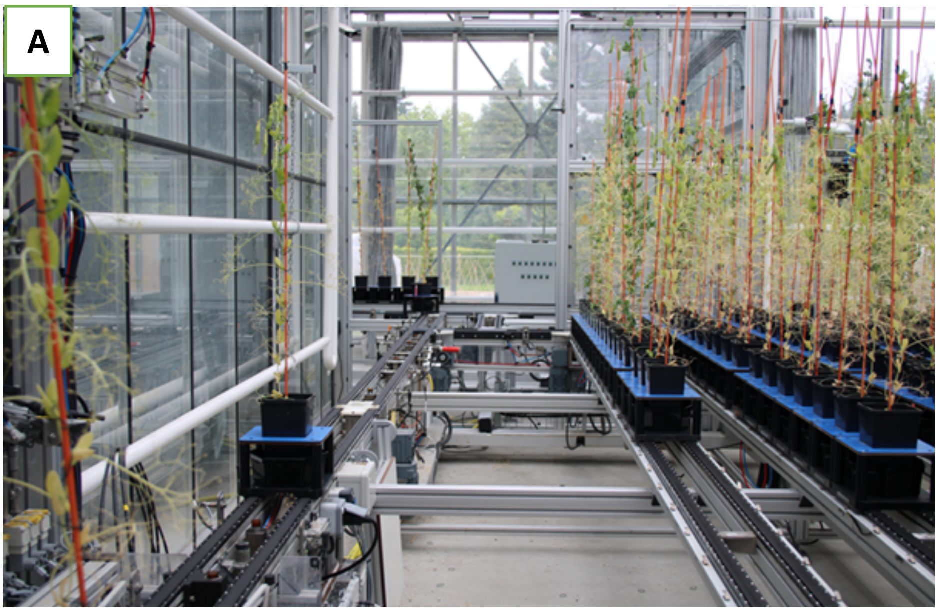

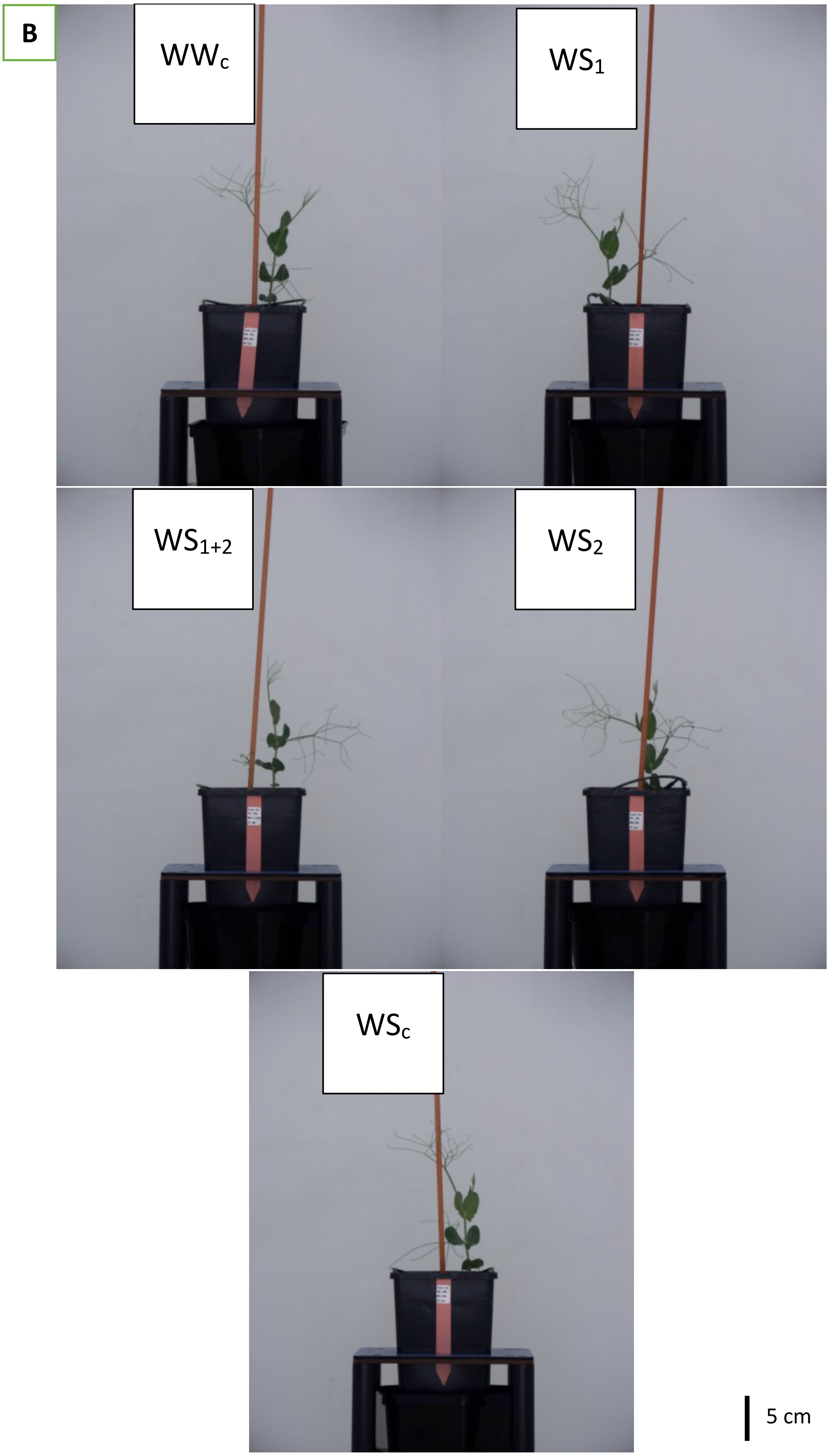
B
WWc
WS1
WS1+2
WS2
WSc
5 cm

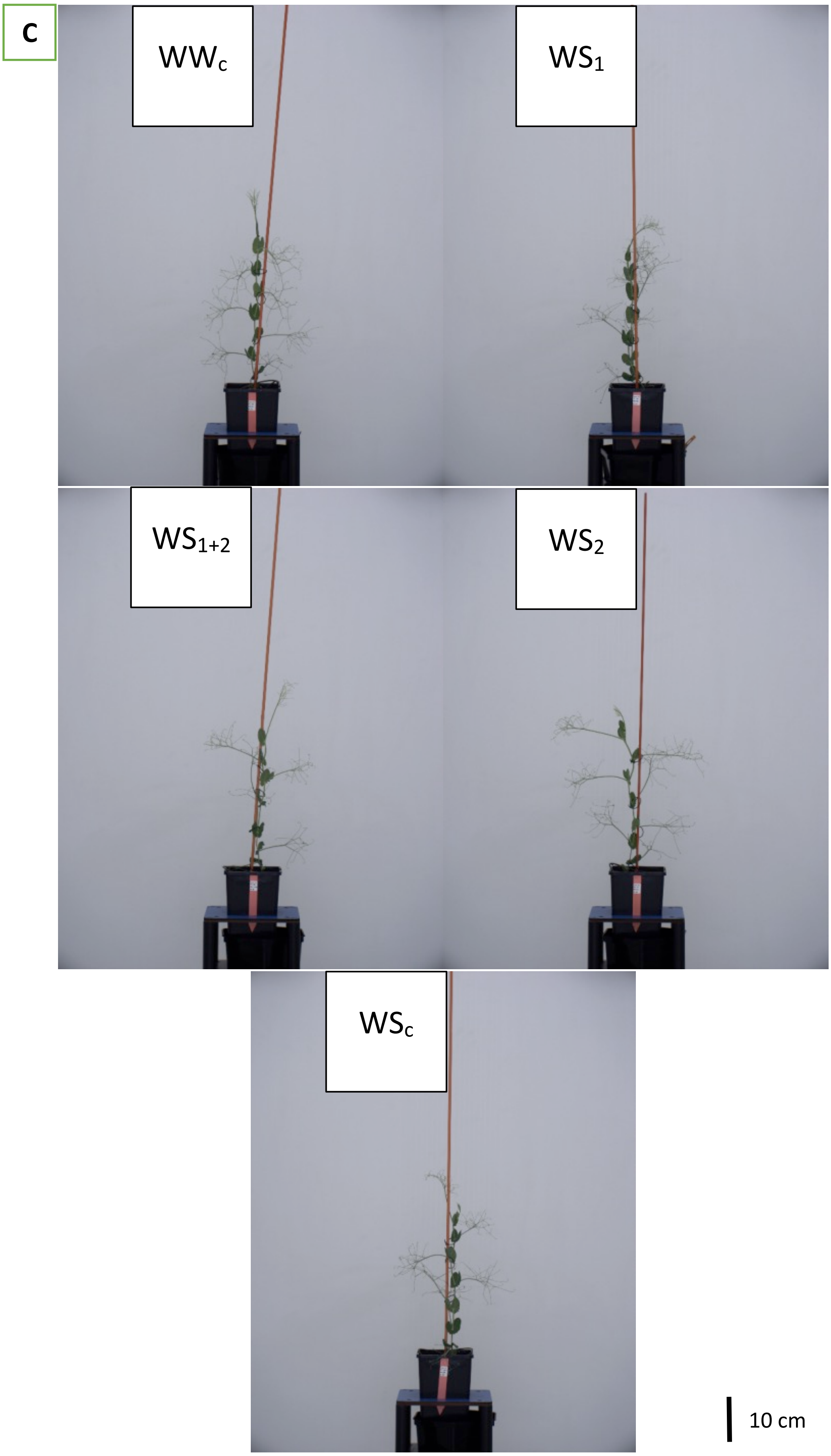
C
WW_c
WS_1
WS_{1+2}
WS_2
WS_c
10 cm

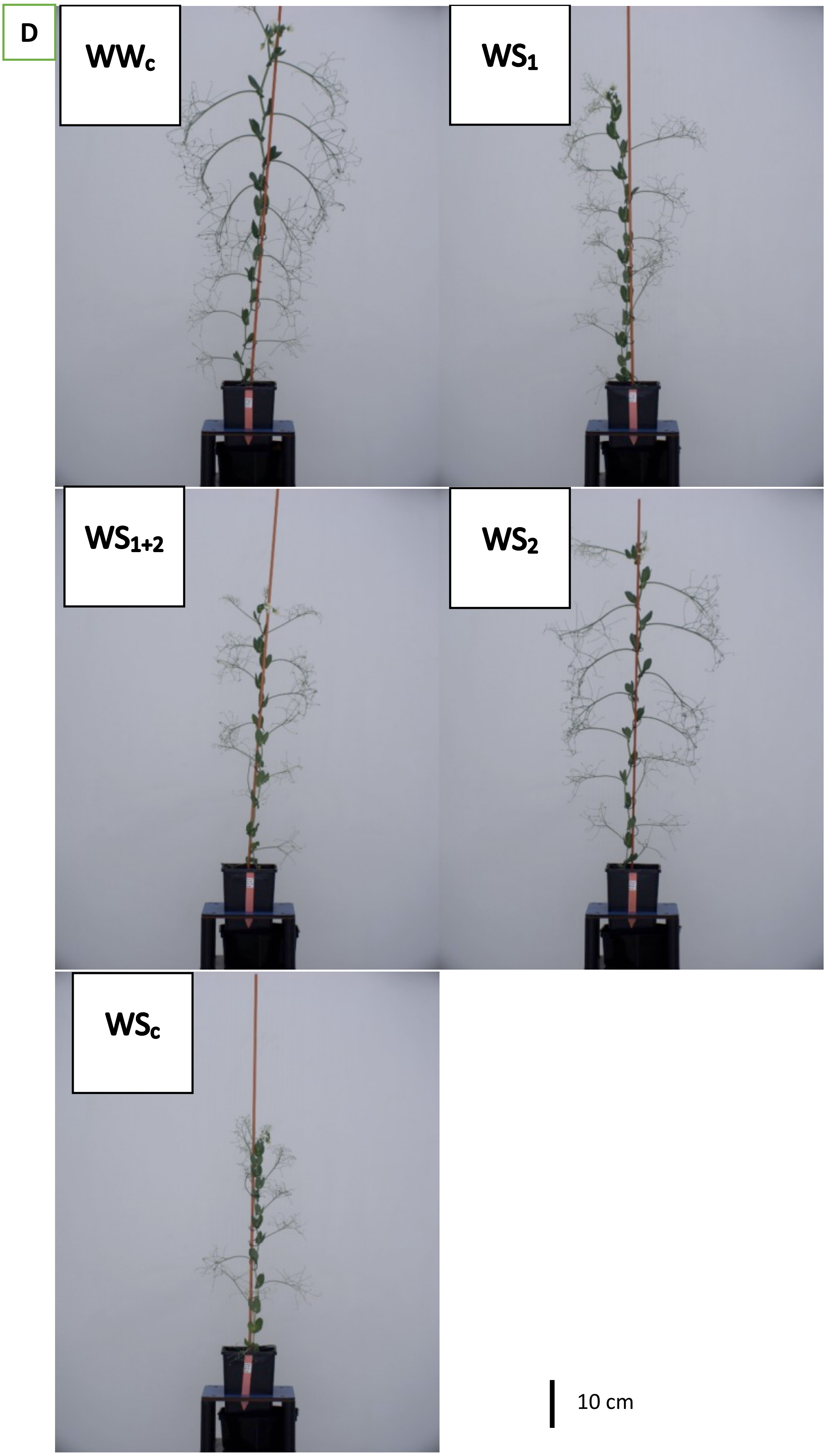
D
WWc
WS1
WS1+2
WS2
WSc
10 cm

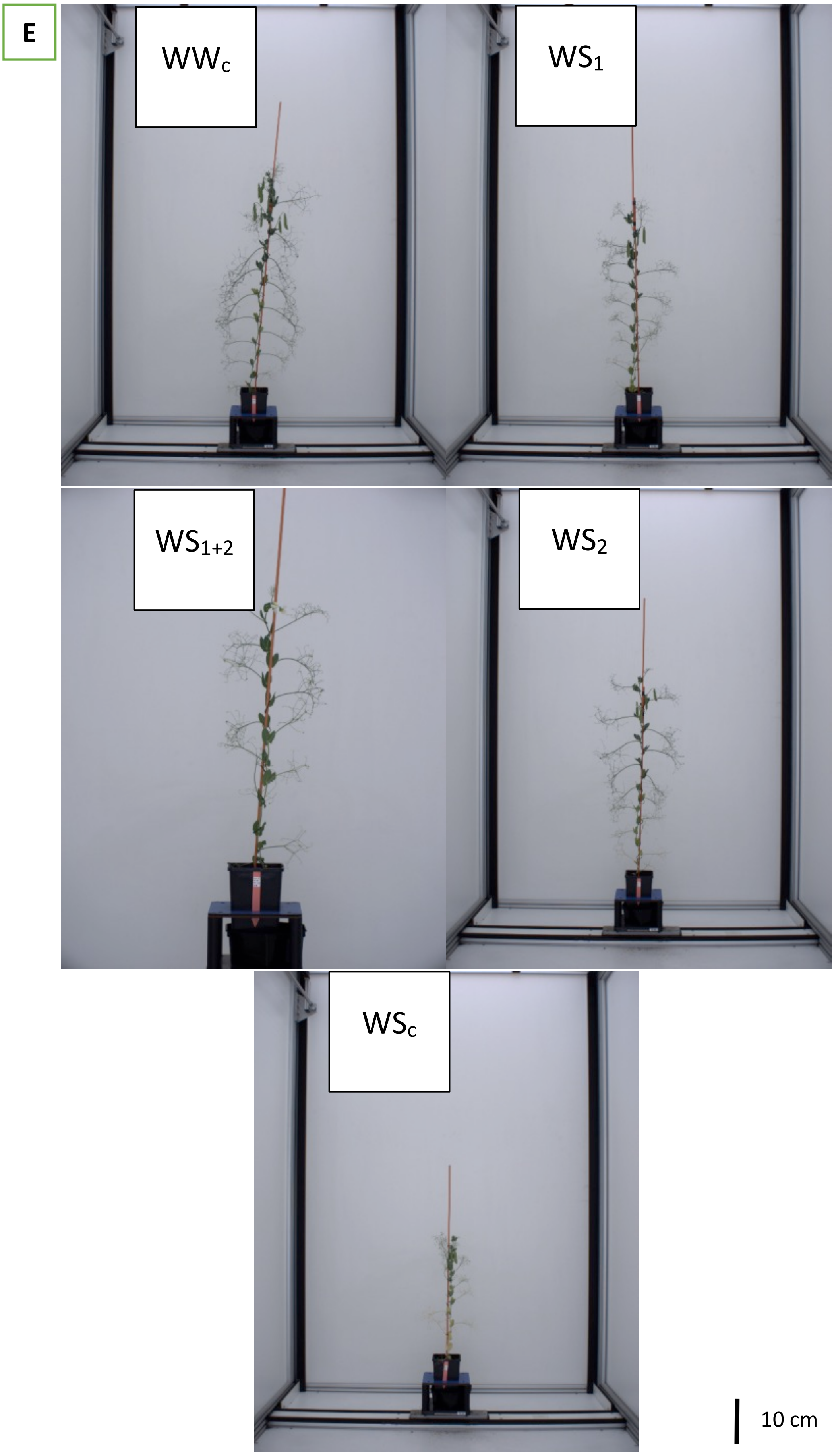
E
WWc
WS1
WS1+2
WS2
WSc
10 cm

Supplementary Material S6

Estimation of "ecophysiological memory" under recurrent stresses, and definition of an ecophysiological imprint of stress memory.

Plant memory to drought has been mainly characterized at the molecular level: genes, metabolites, or proteins (Jacques et al., 2021). Memory genes have been classified according to their pattern of expression throughout the stress (first stress, recovery, and second stress, Ding et al., 2014). The main characteristic of these genes is that their expression during the second stress is different to their expression during a single stress occurring at the same period. That is to say, memory genes can be specifically deregulated during the second stress, or stronger deregulated or inversely deregulated under recurrent stress when compared to a single stress.

By analogy with the molecular memory level, we have characterized ecophysiological memory by identifying changes in ecophysiologival traits were only observed under recurrent stress. In our study, measurements of ecophysiological variables of vegetative single stress at reproductive stage enabled us to determine if typically, the response was only due to vegetative single stress or to cumulative impact of the two stresses.

Moreover, some molecules related to stress response could be stored during recovery period, enabling a stronger or a faster response to water stress. The modulation of either the molecule storage or the protein conformation during recovery is considered as a stress "imprint". By analogy, we have considered that if an ecophysiological variable was improved during recovery, this one could correspond to an "ecophysiological imprint", that could be beneficial for plant recurrent water stress tolerance.
